# rKOMICS: an R package for processing mitochondrial minicircle assemblies in population-scale genome projects

**DOI:** 10.1186/s12859-021-04384-1

**Published:** 2021-09-28

**Authors:** Manon Geerts, Achim Schnaufer, Frederik Van den Broeck

**Affiliations:** 1 Department of Biomedical Sciences, Institute of Tropical Medicine, 2000 Antwerp, Belgium; 2 Institute of Immunology and Infection Research, University of Edinburgh, Edinburgh, EH9 3FL UK; 3 Department of Microbiology, Immunology and Transplantation, Rega Institute for Medical Research, Katholieke Universiteit Leuven, 3000 Leuven, Belgium

**Keywords:** Assembly, Clustering, Minicircles, Sequencing, Kinetoplast, *Leishmania*, *Trypanosoma*, Parasites

## Abstract

**Background:**

The advent of population-scale genome projects has revolutionized our biological understanding of parasitic protozoa. However, while hundreds to thousands of nuclear genomes of parasitic protozoa have been generated and analyzed, information about the diversity, structure and evolution of their mitochondrial genomes remains fragmentary, mainly because of their extraordinary complexity. Indeed, unicellular flagellates of the order Kinetoplastida contain structurally the most complex mitochondrial genome of all eukaryotes, organized as a giant network of homogeneous maxicircles and heterogeneous minicircles. We recently developed KOMICS, an analysis toolkit that automates the assembly and circularization of the mitochondrial genomes of Kinetoplastid parasites. While this tool overcomes the limitation of extracting mitochondrial assemblies from Next-Generation Sequencing datasets, interpreting and visualizing the genetic (dis)similarity within and between samples remains a time-consuming process.

**Results:**

Here, we present a new analysis toolkit—rKOMICS—to streamline the analyses of minicircle sequence diversity in population-scale genome projects. rKOMICS is a user-friendly R package that has simple installation requirements and that is applicable to all 27 trypanosomatid genera. Once minicircle sequence alignments are generated, rKOMICS allows to examine, summarize and visualize minicircle sequence diversity within and between samples through the analyses of minicircle sequence clusters. We showcase the functionalities of the (r)KOMICS tool suite using a whole-genome sequencing dataset from a recently published study on the history of diversification of the *Leishmania braziliensis* species complex in Peru. Analyses of population diversity and structure highlighted differences in minicircle sequence richness and composition between *Leishmania* subspecies, and between subpopulations within subspecies.

**Conclusion:**

The rKOMICS package establishes a critical framework to manipulate, explore and extract biologically relevant information from mitochondrial minicircle assemblies in tens to hundreds of samples simultaneously and efficiently. This should facilitate research that aims to develop new molecular markers for identifying species-specific minicircles, or to study the ancestry of parasites for complementary insights into their evolutionary history.

## Background

Population-scale sequencing projects resort to short-read sequencers such as Illumina's HiSeq or BGI's BGISEQ instruments because they are cost-effective, accurate and supported by a wide range of analysis tools and pipelines [1]. Sequence data in fastq format is typically aligned to a nuclear reference assembly using tools such as BWA [2] or bowtie2 [3] that store alignment details in Sequence Alignment/MAP (SAM) text formats (or its binary equivalent BAM). The aligned sequenced data are then used to call genome-wide variants with e.g. the Genome Analysis Toolkit (GATK) [4] or to detect structural variations [5]. In addition to nuclear sequences, next-generation sequencing platforms produce a high copy number of extranuclear sequences (originating from mitochondria or chloroplasts) when DNA is extracted from whole cells. Such extranuclear sequences can be de novo assembled using tools such as MITObim [6] or NOVOPlasty [7]. This means that both nuclear and extranuclear sequence diversity could be studied during a single next-generation sequencing experiment.

Kinetoplastids are early branching eukaryotes of biomedical and economical relevance with many genera infectious to humans or animals (e.g. *Leishmania* and *Trypanosoma*), plants (e.g. *Phytomonas*) and insects (e.g. *Crithidia*). The advent of whole genome sequencing has revolutionized our biological understanding of these parasitic protozoa. Continuous generation and refinement of reference genome assemblies and annotations [8, 9] provided an important resource for both experimental [10] and natural diversity studies [11–13]. Most notably, population-scale genome studies provided genomic evidence of meiotic-like recombination in presumed clonal pathogens [14–18], revealed the evolutionary and epidemic history of a lethal human pathogen [19, 20] or have set the scene for a new understanding of the evolution and genetics of protozoan parasites [21]. However, while hundreds to thousands of nuclear genomes of parasitic protozoa have been generated and analyzed, information about the diversity, structure and evolution of their mitochondrial genome remains fragmentary.

The mitochondrial genome of unicellular flagellates of the order Kinetoplastida is unique due to its extraordinary complexity and structure compared to the mitochondrial genome of other eukaryotes. It contains a giant network of hundreds to thousands of heterogeneous minicircles (0.5–2.5 kb) interlaced with 20–50 homogeneous maxicircles (20–30 kb), with a topology resembling that of a medieval chain mail [22]. The maxicircles contain four genes (9S rRNA, 12S rRNA, RPS12 and RPS3) that encode rRNA and protein subunits of the mitochondrial ribosome and sixteen genes (ND1, 3, 4, 5, 7, 8 & 9, COI, II & III, Cyb, ATPase6, MURF1 & 2, CR3 & 4) encoding different protein subunits of the respiratory chain and F_1_F_O_ ATP synthase. However, many of the protein-coding maxicircle genes are encrypted (i.e. are cryptogenes) and give rise to pre-mRNAs that require post-transcriptional modification via an elaborate RNA editing process that generates translatable mRNAs. Over 1,200 guide RNAs (gRNAs) are predicted to be responsible for directing this editing process in *T. b. brucei* [23, 24], and the vast majority of these gRNAs are encoded within the minicircles [22]. Minicircles generally consist of a ~ 100 bp conserved sequence region that contains hyper conserved sequences named Conserved Sequence Blocks (CSBs), and a variable region including gRNAs that are flanked by inverted repeats. Trypanosomatids only encode 1–5 different gRNAs per minicircle, which explains the extreme heterogeneity of these molecules in the kinetoplast genome. This RNA editing process represents one of the most striking examples of small RNA regulation of gene expression in eukaryotic cells.

The complex structure of the mitochondrial genome of Kinetoplastids requires a dedicated approach to recuperate a full complement of maxicircles and minicircles from next-generation sequencing datasets. The coding region of the maxicircles displays a highly conserved gene organization with a gene content similar to the mitochondrial genome of other eukaryotes, including yeast and mammals, with the notable exception of the complete absence of tRNA genes. Hence, bioinformatic and downstream phylogenetic analyses of the mitochondrial maxicircle resemble those used for analyzing the mitochondrial genome in higher eukaryotes. Reference maxicircle sequences can be assembled using either short- or long-read sequencing datasets (see [16] for an example on how to sequence, assemble and annotate a complete reference maxicircle genome using paired-end 250-bp Illumina reads). Once a reference maxicircle sequence is available from the species of interest (or from a closely related organism), it can be used in population-scale genome studies for reference-guided assembly (e.g. [25]) or genotyping (e.g. [16]). This approach has been used widely to recover the maxicircles from whole genome sequencing datasets of various protozoan parasites [14, 16, 20, 21].

In contrast to maxicircles, reference-guided assembly or genotyping is not feasible for the minicircles as each individual strain displays an almost unique set of minicircle sequences that occur in variable copy numbers [21, 26]. Hence, minicircles are to be assembled for each parasite sample independently and clustered into Minicircle Sequence Classes (MSCs), here defined as groups of minicircle sequences sharing a given percent identity [21, 24]. Long-read sequencing technologies such as Oxford Nanopore or PacBio should greatly facilitate the assembly process of the highly heterogeneous minicircle populations. These third-generation sequencers are a valid choice when dealing with one or a few samples, as done by [26] for two *Leishmania tarentolae* isolates, but they are not the preferred sequencing technology within the context of a genome diversity study that deals with tens to hundreds of protozoan parasites. Other studies have assembled minicircle sequences from short-read DNA/RNA sequencing in *Leptomonas pyrrhocoris* using a custom seed-extension assembly algorithm [27, 28], or in *Trypanosoma brucei* [24] using the Velvet assembly algorithm [29]. Because fully assembled contigs from circular molecules have duplicated ends, Cooper and colleagues used the CAP3 assembler to search for overlapping ends in contigs that were sliced in two halves [24], also known as the 'circularity test' [30]. Following a similar approach as [24], we recently developed the program KOMICS (which stands for kinetoplast genomics), a python3.7 package that automates the assembly and circularization of mitochondrial minicircles from short-read whole genome sequencing datasets [21]. Assembly is done with MEGAHIT [31] and circularization through a BLAST approach [32] that identifies a sequence in common at the start and the end of a given minicircle contig. Once appropriate assembly parameters are chosen, KOMICS allows the automated recovery of (nearly) complete minicircle genomes for tens to hundreds of samples at a time [21].

While KOMICS overcomes the limitation of acquiring mitochondrial minicircle assemblies, interpreting and visualizing the genetic (dis)similarities of the minicircle sequences within and between (groups of) samples remains a challenging process. Here, we present a new tool named rKOMICS, an R package that allows swift exploration of clusters of minicircle sequences within a set of samples. Our aim is to provide a dedicated bioinformatic tool that streamlines the analyses, visualization and interpretation of minicircle sequence diversity in population-scale genome projects.

## Material and methods

### Implementation

The core features of the rKOMICS package include data aggregation, analyses and visualization that allows to examine, summarize and extract meaningful information from minicircle sequence alignments as provided by KOMICS [21] or a custom bioinformatic pipeline, and from USEARCH cluster format (UC) files as generated by USEARCH [33] or VSEARCH [34]. In addition to storing data files, rKOMICS stores the analyses and visualization results into single list objects that can be called by the user at a later stage. All functions are listed in Table 1, and below we provide a brief description of the main functionalities.

**Table 1:** Summary of commands available in latest development version of rKOMICS

Command	Description
Preprocess	Filters a FASTA file based on minicircle sequence length (specified by a minimum and maximum numeric value) and circularization success (Boolean argument)
msc.length	Calculates and visualizes the length of minicircle sequences
read.uc	Reads and stores USEARCH cluster format (UC) files
msc.uc	Processes clustering results by estimating the number of MSCs and alignment insertions/deletions per minimum percent identity
msc.matrix	Stores clustering results in a matrix where MSCs (rows) are classified as present (1) or absent (0) in each sample (columns)
msc.heatmap	Creates a heatmap that summarizes the presence/absence of MSCs per sample and per group
msc.richness	Summarizes and visualizes the number of MSCs per sample
msc.similarity	Estimates the number and proportion of shared and unique MSCs between groups of samples
msc.subset	Extracts specific MSCs for a given group of samples
msc.pca	Principal Component Analysis to summarize MSC variation for all or a given set of samples
msc.seqs	Retrieves the DNA sequence of a MSC together with all its hit sequences
msc.quality	Checks the quality of the assembly
msc.depth	Calculates the read depth and copy number of the assembled minicircles

Polished minicircle assemblies as obtained with KOMICS or a custom bioinformatic pipeline can be processed with rKOMICS to inspect sequence length distributions, and to extract minicircle sequences that are successfully circularized and of the expected length (usually ~ 700–900 bp for *Leishmania* [26] and ~ 1000–2000 bp for *Trypanosoma* parasites [24]). Once processed, the minicircle sequences from one or more samples are clustered into Minicircle Sequence Classes (MSCs) based on a minimum percent identity (MPI) using USEARCH's or VSEARCH's *cluster-fast* (see below for a typical usage workflow).

The function *msc.uc* reads the output of the clustering analyses (UC file) for each specified MPI into a single list, which will be analyzed automatically to calculate and visualize—per MPI—the number of MSCs, the proportion of perfect alignments (i.e. alignments without any insertion/deletion, but allowing point mutations) and the number of alignment gaps. Gaps are defined by i) the number of insertions/deletions and ii) the length in base pairs of each individual insertion/deletion. It also issues a warning when large gaps (> 500 bp) are found, which points the user to anomalous alignments due to e.g. artificial dimers introduced by the assembly process. This allows the user to make an informed decision about the MPI (or MPI's) that best captures minicircle sequence richness within a (group of) sample(s) while minimizing the number and length of alignment gaps. The function *msc.matrix* reads the output of the clustering analyses (UC file) for each specified MPI and stores it in a matrix where MSCs (rows) are classified as present (1) or absent (0) in each sample (columns). This matrix is used for downstream analyses and visualizations, without relying on the user for data manipulation and reformatting.

We introduce several functions that allow measuring the variation of MSCs within a parasite sample and between parasite samples. The function *msc.richness* returns a measure of minicircle richness per sample, which is simply estimated as the number of MSCs per sample. The function returns a table of estimates per sample and per MPI, as well as a boxplot that shows the minicircle richness in each sample as estimated over a range of MPIs. The function *msc.similarity* returns a measure of minicircle sequence composition within and between groups of samples. Specifically, it estimates the absolute and relative number of MSCs that are unique to each group or shared between two or more groups. The function returns tables and barplots that summarize the number of unique or shared MSCs for each MPI separately or combined over all MPIs.

rKOMICS incorporates multiple methods of visualizations using the ggplot2 R package [35] to plot the foundation of graphs. By adding ggplot2 functions to the rKOMICS visualization functions, the user has direct control over the finishing touches of the graph's appearances. Our package also utilizes sample-specific metadata that allows multi-group data visualizations to facilitate exploratory analysis. The overall data set can be examined using barplots, heatmaps, PCA plots and box plots that are generated for each specified MPI. This makes it possible to visualize population structure and diversity based on minicircle sequence composition.

### A step-by-step tutorial on how to use the (r)KOMICS tool suite

Population-scale genome diversity projects start by extracting DNA from whole cells before generating paired-end whole genome sequencing data that is aligned against a nuclear reference assembly. The aligned sequence data aids genome-wide variant discovery and the detection of structural variations that can be used in downstream population genomic analyses (this will not be discussed here, but see [14–21] for examples). Reads that did not align against the reference genome are putative extranuclear sequences and should be extracted from the alignment file. When a reference maxicircle sequence of the same or a closely related species is available, unaligned reads are mapped against the maxicircle sequence to assist reference-guided assembly or genotyping of the conserved coding region (see [14, 16, 20, 21] for examples).

To obtain de novo assemblies of minicircle and maxicircle sequences per sample, we propose to use the KOMICS software package [21] (https://frebio.github.io/komics/). The input of KOMICS is sequence reads in FASTQ format, and the output is maxicircle and circularized minicircles in FASTA format. Before the assembly process is started, it is recommended to trim reads for high quality and to a maximum length of 150 bp (this is because sequencing quality may decrease with increasing number of cycles). Using the paired-end high-quality trimmed reads, the KOMICS *assemble* command will do the following three major steps:

1. Assemble contigs based on user-specified *k-*mer lengths with MEGAHIT [31]. The optimal *k-*mer length depends on the complexity of the mitochondrial genome, and it will also be different for maxicircles and minicircles. We recommend that the user tries different *k-*mer values in order to—independently—maximize the length of the assembled maxicircle contig and the proportion of circularized minicircle sequences.
2. Extract putative maxicircle sequences using a BLAST [32] approach with several build-in maxicircle sequences of *Leishmania braziliensis* M2904 [21] (available on www.tritrypdb.org), *Trypanosoma lewisi* [36] (GenBank: KR072974.1), *Trypanosoma brucei brucei* (GenBank: M94286.1) and *Trypanosoma equiperdum* STIB842 [37] (GenBank: EU185800.1) as subjects. Identified maxicircle sequences may be further extended using NOVOPlasty [7], circularized using a BLAST approach and annotated using a homology-based search of known maxicircle genes with RATT [38] (see [16] for a detailed example on how to assemble a complete maxicircle sequence using deep-sequencing data of 250 bp paired-end reads). However, depending on sequencing depth and the complexity of the divergent (variable) region of the maxicircle, assembly of a complete maxicircle may prove challenging.
3. Extract minicircle contigs based on the presence of the Conserved Sequence Block 3 (CSB3), a 12-bp minicircle motif that is also known as the universal minicircle sequence and that is highly conserved across all Kinetoplastida species [39]. KOMICS uses the known CSB3 motifs GGGGTTGGTGTA [39] and GGGGTTGATGTA (identified in a subset of minicircles from *T. b. brucei* [24]), and their reverse complements. Note that other less conserved CSB3 motifs can be specified by the user to accommodate the species model being investigated; users should always check for potential alternative CSB3 motifs in the original contigs file using e.g. the MEME Suite [40]. Note that this step will only work for minicircle structures with a single conserved region per minicircle.

Following the assembly and identification of linear mitochondrial minicircle contigs, KOMICS *circularize* uses BLAST as a strategy to identify a sequence that is in common at the start and the end of a given minicircle contig. Whenever an overlap is found, the contig is classified as circular and the duplicated sequence at the start of the contig is removed. Note that the performance of the circularization procedure has not been tested on dimeric or multimeric minicircles. Finally, KOMICS *polish* will (1) reorient each minicircle contig until the CSB3 motifs have the correct direction (GGGGTTG[G/A]TGTA), (2) create a minicircle alignment by rearranging sequences such that the Conserved Sequence Block 1 (CSB1) (GGGCGT[T/G]C) is at the start of each circularized minicircle contig and (3) cluster contigs based on a minimum percent identity using VSEARCH [34]. Note that other less conserved CSB1 and CSB3 motifs can be specified by the user to accommodate the species model being investigated.

**Fig. 1:**
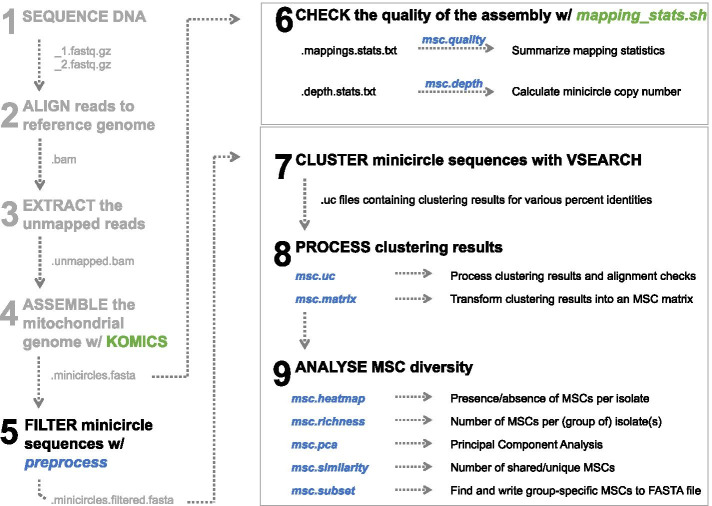
Schematic representation of a typical (r)KOMICS usage workflow. Steps 1–4 are shown in gray and are required bioinformatic analyses prior to the use of rKOMICS. Note that rKOMICS requires minicircle sequence assemblies (step 4) that are either obtained by KOMICS (https://frebio.github.io/komics/) or through a custom bioinformatic pipeline. Steps 5–9 describe the input files required and the results obtained by the rKOMICS software package. Functions implemented in rKOMICS are coloured in blue, while scripts implemented in KOMICS are coloured in green

Following the successful generation of minicircle sequence alignments with KOMICS, the user is recommended to check the quality of the assembly (Fig. 1, step 6). To this end, the original unaligned reads should be mapped against the minicircle sequence assembly using e.g. BWA [2] or bowtie2 [3] and several mapping statistics should be calculated. One useful metric is the proportion of perfect alignments of CSB3-containing reads, which serves as a proxy for the total number of minicircles that were initially present within the DNA sample. We suggest extending the circularized minicircle sequences by 150 bp to avoid clipped reads at either end of the minicircle. To streamline this process, we provide two scripts as part of the KOMICS suite (https://github.com/FreBio/komics/raw/master/komics/fasta_extend.py and https://github.com/FreBio/komics/raw/master/komics/mapping_stats.sh) that will parse SAM files and output the following statistics: number of reads, number of mapped reads, number of properly paired reads, number of reads with mapping quality ≥ 20, number of CSB3-containing reads, number of mapped CSB3-containing reads and number of perfectly aligned CSB3-containing reads (i.e. alignments without any insertion/deletion). In addition, it will provide a results table that gives a detailed overview of the depth statistics per minicircle sequence. These output files can be processed using the rKOMICS scripts *msc.quality* and *msc.depth* to obtain plots and estimates of minicircle copy numbers.

Finally, we propose to use rKOMICS to measure and visualize minicircle sequence diversity in one or more samples. KOMICS will generate assemblies for each sample independently, which can be processed using the rKOMICS command *preprocess* to retain only sequences of the expected length and—desirably—only sequences that are fully circularized (Fig. 1, step 5)*.* Sequences of all samples of interest should be concatenated into a single FASTA file and used for finding MSCs based on a minimum percent identity with VSEARCH using the default parameters (Fig. 1, step 7). rKOMICS then allows the user to read and store the clustering files, as well as to process the files in order to measure and visualize the variation of MSCs within and between parasite samples (Fig. 1, step 8–9) (Table 1).

### (r)KOMICS application example

To show the functionality of rKOMICS, we performed an example analysis using whole-genome sequencing data from a recently published study on the history of diversification of the *Leishmania braziliensis* species complex in Peru [21]. This species complex comprises two closely related species: the lowland and zoonotic *L. braziliensis* parasite circulating in a diverse range of wild mammals in Neotropical rainforests, and the highland anthroponotic *L. peruviana* parasite that is largely endemic to the Pacific slopes of the Peruvian Andes.

A total of 67 *Leishmania* parasites from 47 localities in Peru were cultured and subjected to whole genome sequencing. Sequence data were aligned against a PacBio assembly containing the 35 major chromosomes and a complete circularized maxicircle sequence of 27.69 kb. Using GATK's HaplotypeCaller, the authors called Single Nucleotide Polymorphisms (SNPs) within the major chromosomes and the coding region of the maxicircle. For a full appreciation of the population genomic and phylogenomic analyses using SNPs identified within the nuclear genome and the mitochondrial maxicircle, we direct the reader to the publication by Van den Broeck and colleagues [21].

We extracted the unaligned sequence reads from the BAM files with SAMtools view [41] and converted the BAM file into a FASTQ file using GATK's SamToFastq [4]. Sequence reads were then trimmed with FASTP [42] using the following parameters: -q 30 -u 10 -5 -3 -W 1 -M 30–cut_right–cut_right_window_size 10 –cut_right_mean_quality 30 -l 95 -b 150. Trimmed reads were used for de novo assembly with KOMICS using a *k*-mer sweep strategy with *k*-mer values of 89, 99, 109 and 119. Minicircle contigs were then circularized and polished with KOMICS using the default parameters (see step-by-step tutorial above for details). Sequence reads were aligned to the filtered set of minicircle sequences (both circularized and non-circularized contigs) using SMALT (https://www.sanger.ac.uk/tool/smalt-0/) with a minimum identity threshold of 95%, and processed with the rKOMICS command *msc.quality* to check the quality of the assembly. After inspection of minicircle sequence lengths with the *msc.length* function, individual fasta files were filtered using the *preprocess* function based on a minimum length of 500 bp, a maximum length of 1200 bp and with the circularization parameter set to *true*. The filtered FASTA files containing circularized minicircles per sample were combined into a single file. VSEARCH *–cluster_fasta* was used to identify clusters of minicircle sequences based on a minimum percent identity of 80, 85, 88–100. Clustering results were then processed, transformed, visualized and summarized with the *msc.uc*, *msc.matrix*, *msc.heatmap* and *msc.richness* functions, respectively, after which we analyzed MSC diversity between samples with the *msc.pca* and *msc.similarity* functions.

## Results

We provide a simplified overview of a straightforward analysis of minicircle sequence diversity, while in-depth analyses are provided in the rKOMICS vignette document. Most (but not all) of the results described below were also presented in [21], but we have here re-analyzed the data to showcase the functionality of rKOMICS.

**Fig. 2:**
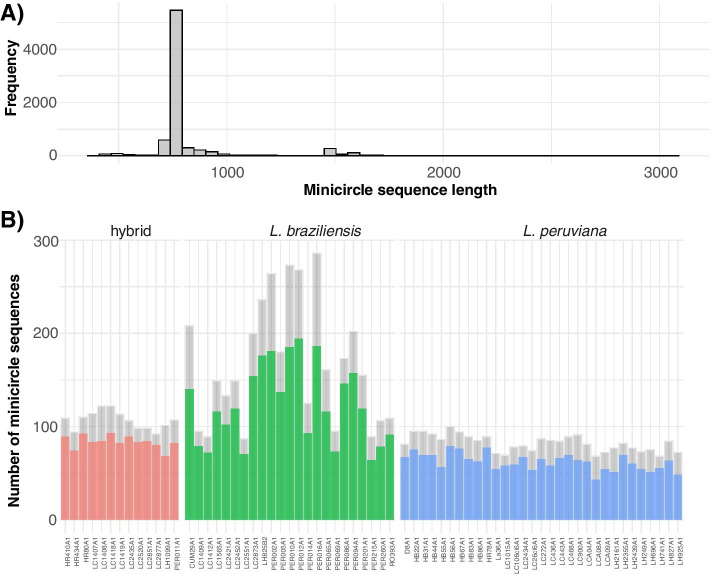
**a** Length distribution of the 7760 assembled minicircles in 67 *Leishmania* isolates, as obtained with rKOMICS function *msc.length*. **b** Gray barplots show the total number of minicircles found per *Leishmania* isolate, and coloured barplots indicate the number obtained after retaining only circularized minicircles of the expected length. Graph is obtained with the rKOMICS function *preprocess*

A combined total of 7760 minicircles were assembled for 67 *Leishmania* isolates. When examining the length distribution of the circularized minicircle sequences using the function *msc.length*, we found that the majority of minicircles (95.2%) were 720–760 bp long, which is within the expected length range of minicircles in *Leishmania* parasites (Fig. 2a). 294 minicircle contigs (4.8%) showed twice this length (1400–1700 bp) (Fig. 2a), which may suggest that these are artificial minicircle dimers introduced by the assembly process, and were subsequently removed. For downstream analyses, we only retained the circularized minicircles of the expected length (720–760 bp) using the function preprocess (Fig. 2b; coloured barplots), resulting in a final set of 5849 minicircles. When examining the quality of the assembly by alignment of reads to the assembled minicircles using the function msc.quality, we found that on average 77% of all mapped reads aligned with a mapping quality larger than 20 and on average 84% aligned in proper pairs. On average 93% of all CSB3-containing reads aligned against the assembled minicircle contigs and 88.5% aligned perfectly, suggesting that KOMICS was able to retrieve a large proportion of the minicircle classes.

**Fig. 3:**
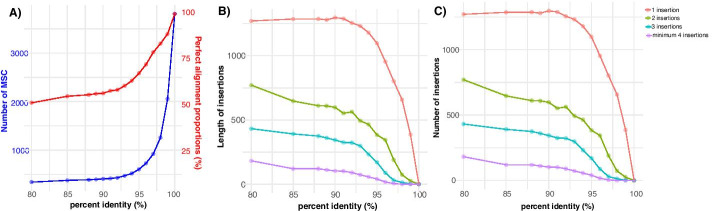
**a** Number of MSCs (blue) and proportion of perfect alignments (red) as obtained following clustering analyses for a range of percent identities. **b**, **c** Length and number of insertions in MSC alignments following clustering analysis for a range of percent identities. These graphs are obtained with the rKOMICS function *msc.uc*

We used the function msc.uc to examine the combined number of minicircle sequence classes (MSCs) (based on overall identity) across all 67 isolates, and identified a total of 3811 MSCs at 100% identity. This number decreased sharply to 918 MSCs at 97% identity and 603 MSCs at 95% identity (Fig. 3a). The proportion of perfectly aligned minicircle sequences (i.e. alignments without any insertion/deletion) during the clustering process decreased from 100% (only perfect alignments) at 100% identity to 79% (79% of the alignments were perfect) at 97% identity and 68% at 95% identity (Fig. 3a). While insertions were mostly 1 bp long (Fig. 3b), the number of insertions per alignment increased with decreasing percent identity (Fig. 3c). Most notably, below 97% identity, we found a steady increase in alignments with 3 or 4 insertions (Fig. 3c). Similar results were obtained for deletions (results not shown). Hence, we decided to focus most of our downstream analyses at the 97% identity threshold, as this would capture sufficient minicircle sequence classes (Fig. 3a) while minimizing the number of alignment gaps (Fig. 3b, c).

**Fig. 4:**
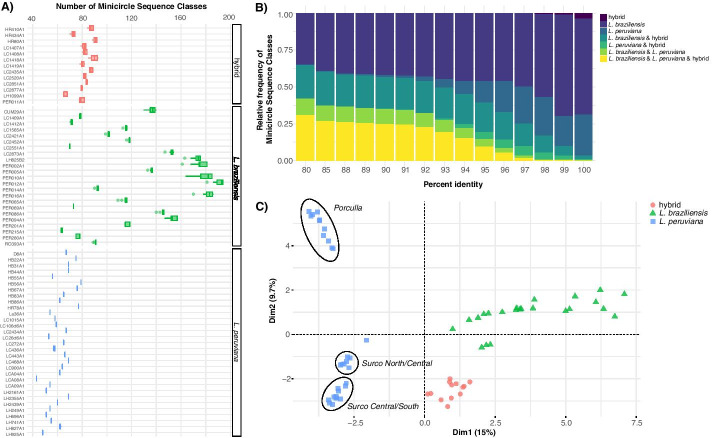
**a** Boxplot as obtained with rKOMICS function *msc.richness* summarizing the number of Minicircle Sequence Classes (MSCs) for various percent identity thresholds (80%, 85%, 87–100%). **b** Barplot obtained with rKOMICS function *msc.similarity* showing the proportion of minicircle sequence classes that are unique or shared between *L. braziliensis*, *L. peruviana* and their hybrids, for each percent identity threshold used during the clustering analyses. **c** Principal Component Analysis as obtained with function *msc.pca* based on sequence similarity between MSCs at 97% identity

Focusing on the results at 97% identity, we observed that the Andean and near-clonal *L. peruviana* parasites harbored substantially less MSCs (mean = 62 MSCs per isolate) compared to the Amazonian and recombining *L. braziliensis* parasites (mean = 124 MSCs per isolate) (Fig. 4a). Using the function msc.similarity, we found that 48.9% and 25.9% of the MSCs were unique to *L. braziliensis* and *L. peruviana*, respectively, while hybrid *L. braziliensis x L. peruviana* parasites shared MSCs with both parents (Fig. 4b). This confirms that hybrid parasites inherited minicircles from both *Leishmania* parental species, a phenomenon that is described in more detail in [21]. Principal Component Analysis based on minicircle sequence similarity (i.e. MSC presence/absence per isolate) separated *L. braziliensis* from *L. peruviana* on the first axis and three *L. peruviana* populations on the second axis (Fig. 4c). The three *L. peruviana* populations correspond to the Porculla lineage that circulates in the tropical deciduous forests of Peru, and the two Surco lineages that circulate in desert shrubland on the Pacific Coast (Surco North/Central and Surco Central/South) (see [21] for more details on the ancestry of these parasites). Hybrids did not cluster with either parental species, in contrast to what was observed for the uniparentally inherited kinetoplast [21], but instead occupied an intermediate position between *L. braziliensis* and the *L. peruviana* Surco Central/South lineage (Fig. 4b), again consistent with mixing of the parental minicircle populations [21].

## Conclusion

As shown by the (r)KOMICS step-by-step tutorial and application example, the process of extracting and analyzing the mitochondrial genome of Kinetoplastids is challenging, requiring dedicated bioinformatic tools. With rKOMICS, we provide a versatile tool to explore and compare minicircle sequence diversity in tens to hundreds of samples. Our tool is applicable to all 30 + Kinetoplastid genera, including the 27 trypanosomatid genera, with recent studies indicating that the actual diversity of the Kinetoplastea is much higher than previously anticipated [43]. We envision that the (r)KOMICS tool suite will be integrated in bioinformatic pipelines that aim to examine the ancestry of these parasitic protozoans based on a joint analysis of the complete nuclear and mitochondrial genome. One limitation of our implementation is that MSC classification is solely based on sequence similarity and does not consider gRNA genes. However, gRNA annotation requires detailed knowledge of edited maxicircle mRNA sequences [24], which vary between species and isolates, and this knowledge is usually not available. Hence, we believe that MSC classification based on conservation of sequence identity is most appropriate for the purpose and applicability of our software package. More-over, because rKOMICS analyzes and summarizes UC cluster format files, it should also become applicable to metagenomic studies that aim to quickly examine sequence diversity and composition within one or more environmental samples.

### Availability and requirements

- Project name: rKOMICS
- Project home page: https://CRAN.R-project.org/package=rKOMICS
- Operating system(s): Platform independent
- Programming language: R
- Other requirements: None
- License: LGPL

Any restrictions to use by non-academics: LGPL license, open source.

## Data Availability

The minicircle sequence alignment file that support the findings of this study are available at https://github.com/FreBio/komics/tree/master/data/LBRA.all.circularized.minicircles.fasta and were generated from the following study: Van den Broeck F, Savill NJ, Imamura H, Sanders M, Maes I, Cooper S, et al. Ecological divergence and hybridization of Neotropical *Leishmania* parasites. Proc Natl Acad Sci USA. 2020;117.

## Abbreviations

bp: Base pair(s)
kbp: Kilo base pair(s)
KOMICS: Kinetoplast genomics
UC: USEARCH cluster
BGI: Beijing Genomics Institute
BWA: Burrows–Wheeler alignment
SAM: Sequence alignment/map
BAM: Binary alignment map
GATK: Genomic analysis toolkit
DNA: Deoxyribonucleic acid
MITObim: Mitochondrial baiting and iterative mapping
RNA: Ribonucleic acid
mRNA: Messenger RNA
gRNA: Guide RNA
T.b.: *Trypanosoma brucei*
MSC: Minicircle sequence class
PCA: Principal component analysis
MPI: Minimum percent identity
CSB: Conserved sequence block
L.: Leishmania
SNP: Single nucleotide polymorphism

## References

[1] Goodwin S, McPherson JD, McCombie WR (2016). Coming of age: ten years of next-generation sequencing technologies. Nat Rev Genet.

[2] Li H, Durbin R (2010). Fast and accurate long-read alignment with Burrows–Wheeler transform. Bioinformatics.

[3] Langmead B, Trapnell C, Pop M, Salzberg SL (2009). Ultrafast and memory-efficient alignment of short DNA sequences to the human genome. Genome Biol.

[4] DePristo MA, Banks E, Poplin R, Garimella KV, Maguire JR, Hartl C (2011). A framework for variation discovery and genotyping using next-generation DNA sequencing data. Nat Genet.

[5] Cameron DL, Di Stefano L, Papenfuss AT (2019). Comprehensive evaluation and characterisation of short read general-purpose structural variant calling software. Nat Commun.

[6] Hahn C, Bachmann L, Chevreux B (2013). Reconstructing mitochondrial genomes directly from genomic next-generation sequencing reads—a baiting and iterative mapping approach. Nucleic Acids Res.

[7] Dierckxsens N, Mardulyn P, Smits G (2017). NOVOPlasty: de novo assembly of organelle genomes from whole genome data. Nucleic Acids Res.

[8] Berriman M, Ghedin E, Hertz-Fowler C, Blandin G, Renauld H, Bartholomeu DC (2005). The genome of the African trypanosome *Trypanosoma brucei*. Science.

[9] Peacock CS, Seeger K, Harris D, Murphy L, Ruiz JC, Quail MA (2007). Comparative genomic analysis of three Leishmania species that cause diverse human disease. Nat Genet.

[10] Tihon E, Imamura H, Van den Broeck F, Vermeiren L, Dujardin J-C, Van Den Abbeele J (2017). Genomic analysis of isometamidium chloride resistance in *Trypanosoma congolense*. Int J Parasitol Drugs Drug Resist.

[11] Cuypers B, Lecordier L, Meehan CJ, Van den Broeck F, Imamura H, Büscher P (2016). Apolipoprotein L1 variant associated with increased susceptibility to trypanosome infection. MBio.

[12] Cuypers B, Van den Broeck F, Van Reet N, Meehan CJ, Cauchard J, Wilkes JM (2017). Genome-wide SNP analysis reveals distinct origins of *Trypanosoma evansi* and *Trypanosoma equiperdum*. Genome Biol Evol.

[13] Domagalska MA, Imamura H, Sanders M, Van den Broeck F, Bhattarai NR, Vanaerschot M (2019). Genomes of Leishmania parasites directly sequenced from patients with visceral leishmaniasis in the Indian subcontinent. PLoS Negl Trop Dis.

[14] Rogers MB, Downing T, Smith BA, Imamura H, Sanders M, Svobodova M (2014). Genomic confirmation of hybridisation and recent inbreeding in a vector-isolated leishmania population. PLoS Genet.

[15] Tihon E, Imamura H, Dujardin J-C, Van Den Abbeele J, Van den Broeck F (2017). Discovery and genomic analyses of hybridization between divergent lineages of *Trypanosoma congolense*, causative agent of Animal African trypanosomiasis. Mol Ecol.

[16] Van den Broeck F, Tavernier LJM, Vermeiren L, Dujardin JC, Van Den Abbeele J (2018). Mitonuclear genomics challenges the theory of clonality in *Trypanosoma congolense*: reply to Tibayrenc and Ayala. Mol Ecol.

[17] Schwabl P, Imamura H, Van den Broeck F, Costales JA, Maiguashca-Sánchez J, Miles MA (2019). Meiotic sex in Chagas disease parasite *Trypanosoma cruzi*. Nat Commun.

[18] Inbar E, Shaik J, Iantorno SA, Romano A, Nzelu CO, Owens K (2019). Whole genome sequencing of experimental hybrids supports meiosis-like sexual recombination in leishmania. PLoS Genet.

[19] Imamura H, Downing T, Van den Broeck F, Sanders MJ, Rijal S, Sundar S (2016). Evolutionary genomics of epidemic visceral leishmaniasis in the Indian subcontinent. Elife.

[20] Franssen SU, Durrant C, Stark O, Moser B, Downing T, Imamura H (2020). Global genome diversity of the *Leishmania donovani* complex. Elife.

[21] Van den Broeck F, Savill NJ, Imamura H, Sanders M, Maes I, Cooper S (2020). Ecological divergence and hybridization of neotropical Leishmania parasites. Proc Natl Acad Sci USA.

[22] Lukes J, Guilbride DL, Votýpka J, Zíková A, Benne R, Englund PT (2002). Kinetoplast DNA network: evolution of an Improbable structure. Eukaryot Cell.

[23] Koslowsky D, Sun Y, Hindenach J, Theisen T, Lucas J (2014). The insect-phase gRNA transcriptome in *Trypanosoma brucei*. Nucleic Acids Res.

[24] Cooper S, Wadsworth ES, Ochsenreiter T, Ivens A, Savill NJ, Schnaufer A (2019). Assembly and annotation of the mitochondrial minicircle genome of a differentiation-competent strain of *Trypanosoma brucei*. Nucleic Acids Res.

[25] Wu J, Liu B, Cheng F, Ramchiary N, Choi SR, Lim YP (2012). Sequencing of chloroplast genome using whole cellular DNA and solexa sequencing technology. Front Plant Sci.

[26] Simpson L, Douglass SM, Lake JA, Pellegrini M, Li F (2015). Comparison of the mitochondrial genomes and steady state transcriptomes of two strains of the trypanosomatid parasite, leishmania tarentolae. PLoS Negl Trop Dis.

[27] Gerasimov ES, Gasparyan AA, Afonin DA, Zimmer SL, Kraeva N, Lukeš J (2021). Complete minicircle genome of Leptomonas pyrrhocoris reveals sources of its non-canonical mitochondrial RNA editing events. Nucleic Acids Res.

[28] Gerasimov ES, Gasparyan AA, Litus IA, Logacheva MD, Kolesnikov AA (2017). Minicircle kinetoplast genome of insect trypanosomatid leptomonas pyrrhocoris. Biochemistry.

[29] Zerbino DR, Birney E (2008). Velvet: algorithms for de novo short read assembly using de Bruijn graphs. Genome Res.

[30] Jørgensen TS, Xu Z, Hansen MA, Sørensen SJ, Hansen LH (2014). Hundreds of circular novel plasmids and DNA elements identified in a rat cecum metamobilome. PLoS ONE.

[31] Li D, Liu CM, Luo R, Sadakane K, Lam TW (2015). MEGAHIT: an ultra-fast single-node solution for large and complex metagenomics assembly via succinct de Bruijn graph. Bioinformatics.

[32] Altschul SF, Gish W, Miller W, Myers EW, Lipman DJ (1990). Basic local alignment search tool. J Mol Biol.

[33] Edgar RC (2010). Search and clustering orders of magnitude faster than BLAST. Bioinformatics.

[34] Rognes T, Flouri T, Nichols B, Quince C, Mahé F (2016). VSEARCH: A versatile open source tool for metagenomics. PeerJ.

[35] Ginestet C (2011). Ggplot2: elegant graphics for data analysis: book reviews. J R Stat Soc Ser A Stat Soc.

[36] Lin R-H, Lai D-H, Zheng L-L, Wu J, Lukeš J, Hide G (2015). Analysis of the mitochondrial maxicircle of *Trypanosoma lewisi*, a neglected human pathogen. Parasites Vectors..

[37] Lai D-H, Hashimi H, Lun Z-R, Ayala FJ, Lukes J (2008). Adaptations of Trypanosoma brucei to gradual loss of kinetoplast DNA: *Trypanosoma equiperdum* and *Trypanosoma evansi* are petite mutants of *T. brucei*. Proc Natl Acad Sci USA.

[38] Otto TD, Dillon GP, Degrave WS, Berriman M (2011). RATT: rapid annotation transfer tool. Nucleic Acids Res.

[39] Ray DS (1989). Conserved sequence blocks in kinetoplast minicircles from diverse species of trypanosomes. Mol Cell Biol.

[40] Bailey TL, Johnson J, Grant CE, Noble WS (2015). The MEME suite. Nucleic Acids Res.

[41] Li H, Handsaker B, Wysoker A, Fennell T, Ruan J, Homer N (2009). The sequence alignment/map format and SAMtools. Bioinformatics.

[42] Chen S, Zhou Y, Chen Y, Gu J (2018). fastp: an ultra-fast all-in-one FASTQ preprocessor. Bioinformatics.

[43] d’Avila-Levy CM, Boucinha C, Kostygov A, Santos HLC, Morelli KA, Grybchuk-Ieremenko A (2015). Exploring the environmental diversity of kinetoplastid flagellates in the high-throughput DNA sequencing era. Mem Inst Oswaldo Cruz.

